# The effect of low-temperature straw-degrading microbes on winter wheat growth and soil improvement under straw return

**DOI:** 10.3389/fmicb.2024.1391632

**Published:** 2024-07-11

**Authors:** Yuanyuan Huang, Yuanyuan Yan, Yang Ma, Xiang Zhang, Qian Zhao, Mingxin Men, Yali Huang, Zhengping Peng

**Affiliations:** ^1^Key Laboratory of Farmland Eco-Environment of Hebei/ College of Resources and Environmental Sciences, Hebei Agricultural University, Baoding, China; ^2^State Key Laboratory of North China for Crop Improvement and Regulation/North China Key Laboratory for Crop Germplasm Resources of Education Ministry, Hebei Agricultural University, Baoding, China; ^3^Biology Institute, Hebei Academy of Sciences, Shijiazhuang, China; ^4^Laboratory of Crop Germplasm Resources of Hebei Province/ College of Agronomy, Hebei Agricultural University, Baoding, China; ^5^School of Environmental Science and Engineering, Hebei University of Science and Technology, Shijiazhuang, China

**Keywords:** straw return, straw-degrading microbes, soil textures, yield, soil microbial community

## Abstract

The application of straw-degrading microbes (SDMs) with straw returned to the field is an effective measure to improve soil quality, increase yield, and maintain soil microorganisms. However, the utilization of SDMs in winter in north China is limited by the poor effects at low temperatures. This study investigated the effects of a new compound SDM, including a novel low-temperature fungus *Pseudogymnoascus* sp. SDF-LT, on winter wheat yield, soil improvement, and soil microbial diversity. A 2-year field experiment was conducted in two different soil textures of wheat–maize rotation fields with full corn straw return and application of SDMs at an amount of 67.5 kg hm^−2^. After 2 years of continuous application of SDMs, the winter wheat yield increased significantly, reaching 9419.40 kg hm^−2^ in Ningjin (NJSDM) and 9107.25 kg hm^−2^ in Mancheng (MCSDM). The soil properties have been significantly improved compared with the single straw return group, especially the sandy loam soil, whose quality is relatively low. The analysis of soil microbial diversity showed that SDMs significantly reduced the Chao1, Shannon, Simpson, and observed species of the sandy loam soil in the MCSDM group. The Simpson and Shannon indexes of fungi diversity in the two experimental sites were significantly increased by SDMs. The negative correlation of fungi increased from 47.1 to 48.85% in the SDM groups. The soil-dominant microbes changed in the SDM groups, in which the interactions between microbes were enhanced. These results suggested that the SDMs changed the the soil microbial community structure and its diversity and complexity, which is beneficial for crop growth. Our study provided sufficient evidence for the utilization of low-temperature SDMs with straw return in cold winter, which plays a role in soil improvement, especially for low-quality soils, to increase crop yield.

## Introduction

China is a large agricultural country that has produced more than 819 million tons of crop residues in 2021 (Zhao et al., 2019; Bai et al., 2023). The winter wheat–summer maize rotation system is the largest planting mode in north China that produces more than 500 million tons of straw annually (Islam et al., 2023). The straw resources are excessive with the increase in crop yield resulting in potential environmental problems unless the straws are recycled appropriately (Wang et al., 2022). Straw burning and disposal are forbidden in cases of adverse effects on agriculture and environment, which is in line with the concept of sustainable agricultural development (Hong et al., 2016). Therefore, the outstanding contribution of straw return to the agricultural ecological environment has gradually emerged.

Straw returning technology is currently the most effective and greenish way to utilize straw resources, which have been improved to increase soil organic carbon, maintain soil quality and health, promote crop yield, and improve plant immunity (Tang et al., 2019; Hao et al., 2022; Yue et al., 2023). Straw is rich in organic matter, nitrogen, phosphorus, potassium, and other trace elements, which makes it an ideal fertilizer for crop growth. However, the main components of straw, including lignin, cellulose, and hemicellulose, are all aromatic polymer compounds with complex three-dimensional structures that are difficult to degrade in the natural environment, resulting in limited effects of the application of *in situ* straw return (Bilal et al., 2017; Gong et al., 2020). Furthermore, the high C/N ratio of the returned straws could result in N immobilization, leading to a lack of available nitrogen (AN) in the soil, which needs to be compensated by additional N application, affecting crop growth in turn. Additionally, slowly degrading straws provide a more habitable place for pathogens and eggs, thus causing aggravation of crop pests and diseases (Li et al., 2016; Liu et al., 2016).

There are abundant microorganisms that can produce cellulase and degrade straws. Microorganisms play a crucial role in natural straw degradation. However, only 200 microorganisms have been found and utilized (Taha et al., 2015). Fungi are most widely applied among bacteria, fungi, and actinomycetes that could degrade cellulose. This is because of the ability of fungi to produce strong enzymes and deep penetration of the hyphae into the plant stratum corneum (Gao et al., 2020). The compound microbes commonly function better to degrade cellulose and lignin resulting in an increased straw degradation rate (Chen et al., 2019; Chu et al., 2021). Exogenous application of compound bacterial systems plays a positive role in the abundance and diversity of the soil-dominant bacteria, thereby changing the indigenous microbial community structure of bacteria and fungi (Chen et al., 2021; Nigussie et al., 2021). Previous research found that adding exogenous lignocellulose decomposition microbes changed the indigenous soil microbial community structure and increased crop yield, and it also increased the functional abundance of soil microorganisms (Phukongchai et al., 2022; Wang et al., 2023).

Most of the commercial straw-degrading microbes (SDMs) are functional at warm temperature. However, agricultural land is located broadly in the temperate zone that harbors a cold winter, during which the long-term low temperature of the earth is fatal for SDMs. Many SDMs cease growth and metabolism in winter, resulting in the loss of their straw degradation function. In contrast, the low-temperature straw decomposition bacteria are unconstrained by cold temperatures. Thereby, low-temperature SDMs fully utilize their superior cellulose digestion abilities under natural conditions, regardless of the effects of low and warm temperatures, and save energy and production costs during cultivation (Huang et al., 2020). Thus, we have previously isolated a fungus from the wheat–maize rotation areas that degraded corn straw effectively under 10°C. In this study, we aimed to investigate the effect of compound microbial systems on soil quality, wheat yield, and soil microbial community structure in different soil textures to support the further application of the low-temperature SDMs.

## Materials and methods

### Experimental site description

The experimental sites were located in Ningjin County (NJ) of Xintai City and Mancheng County (MC) of Baoding City in Hebei Province of China (37°36′–38°43′N, 114°43′–115°6′E). These regions had typical temperate continental monsoon climates. The average annual temperature is 12.82–12.90°C. The mean annual precipitation is 499.0–546.5 mm, mainly occurring in June, July, and August. The tested soil was classified into two types: medium loam soil (MLS) in NJ and sandy loam soil (SLS) in MC. The general chemical properties of soil are shown in Table 1.

**Table 1:** General chemical properties of soil.

Experiment site	Soil texture	TN (g kg^−1^)	OM (g kg^−1^)	AN (mg kg^−1^)	AP (mg kg^−1^)	AK (mg kg^−1^)	pH
NJ	MLS	1.80 ± 0.03	29.89 ± 0.89	124.08 ± 0.53	31.10 ± 0.45	249.00 ± 0.24	8.15 ± 0.08
MC	SLS	1.56 ± 0.02	29.34 ± 1.34	145.78 ± 15.59	27.49 ± 0.51	168.00 ± 0.71	8.32 ± 0.10

TN, Total nitrogen; OM, organic matter; AN, alkaline hydrolysis N; AK, available K; AP, available P. Data in this table are represented by mean ± standard deviation (*n* = 3).

### Experimental design

This study was initiated in 2021 and conducted in a rotation system of winter wheat and summer maize, in which winter wheat was planted in October and harvested in June of the next year, and summer maize was directly seeded and grown from June to October. The basal compound fertilizer was applied before wheat sowing, which contained 280.5 kg hm^−2^ N, 132 kg hm^−2^ P_2_O_5_, and 60 kg hm^−2^ K_2_O. Topdressing was applied at the regreening stage of wheat with 375 kg hm^−2^ urea. The corn straws were chopped into pieces of 3–5 cm (length) before mulching on the surface of the soil, followed by fully retained by rotary tilling (depth of 0–10 cm). The planting density of wheat (variety No. 1 of Malan) was 262.50 kg hm^−2^ with a row spacing of 20 cm. The crop cultivation and farming management were in accordance with the local conventional cultivation practices. The straw return treatment without a straw-degrading agent served as a control check (CK), and the straw return with the application of the SDMs with an amount of 67.5 kg hm^−2^ was set as the experimental groups. The experiments were performed in NJ and MC in the same year, with a randomized block design with three replicates for three plots, and each plot area was 100 m^2^ (10.0 m × 10.0 m).

The SDMs were composed of *Pseudogymnoascus* sp. SDF-LT and *Bacillus licheniformis*. The effective number of viable microbes was 100 million g^−1^. The author previously isolated the fungus *Pseudogymnoascus* sp. SDF-LT with a strong cellulose degradation ability at 10°C, whose registration number is OM 304630 on NCBI.

### Soil sampling and analysis

A previous study on the rate of straw degradation by the SDMs was performed in NJ with the string-bag method, which showed a higher straw degradation rate until the wheat filling stage (Supplementary Figure S1). Since the filling stage is important for wheat yield, the soil samples were collected immediately after the wheat filling stage. For each plot, five cores (0–20 cm in depth and 5 cm in diameter) were randomly selected to collect the soil samples. All soil samples were collected into separate sterile plastic bags and stored temporarily in an icebox before being examined in the lab. After removing leaf litter, rocks, and roots, the soil samples were divided into two subsamples: one was stored at −80°C for the determination of microbial diversity, and the other was air-dried for the determination of chemical properties.

Soil pH was determined with a water: soil ratio of 2.5:1 (V/W) using a pH meter (Luo et al., 2017). Soil organic matter (SOM) was determined using the volumetric method. Soil total nitrogen (TN) was measured by the micro-Kjeldahl method. Soil AN was determined by the alkali-hydrolysis diffusion method. Soil available phosphorous (AP) was extracted with 0.5 M NaHCO_3_ solution and analyzed by the Mo-Sb colorimetric method. Soil available potassium (AK) was determined by flame photometry (FP640, Shanghai, China).

During the winter wheat harvest, spike number, grain number, 1000-grain weight, and yield were measured.

### High-throughput sequencing

The microbial DNA was extracted from the 12 soil samples using the OMEGA Soil DNA Kit (M5635-02, Omega Bio-Tek, Norcross, GA, United States), followed by the quantity and quality examination using a NanoDrop NC2000 spectrophotometer (ThermoFisher Scientific, Waltham, MA, United States) and agarose gel electrophoresis, respectively. The V3–V4 regions of the 16S rRNA genes of the soil bacteria were amplified using primers 338F (5′-ACTCCTACGGGAGGCAGCAG-3′) and 806R (5′-GGAC TACHVGGGTWTCTAAT-3′). Fungal ITS rRNA genes were amplified with primers ITS1F (5′-CTTGGTCATTTAGAGGAAGTAA-3′) and ITS2R (5′-GCTGCGTTCTTCATCGATGC-3′). Sample-specific 7-bp barcodes were incorporated into the primers for multiplex sequencing. The PCR amplicons were purified with Vazyme VAHTS DNA Clean Beads (Vazyme, Nanjing, China) and quantified using the Quant-iT PicoGreen dsDNA Assay Kit (Invitrogen, Carlsbad, CA, United States). The amplicons were then pooled in equal amounts and pair-end (2,250 bp) sequenced with NovaSeq 6,000 SP Reagent Kit (500 cycles) on the Illumina NovaSeq platform at Shanghai Personal Biotechnology Co., Ltd. (Shanghai, China).

### Bioinformatic analysis

The raw sequence data were demultiplexed using the demux plugin, followed by cutting primers with the cutadapt plugin (Martin, 2011). Then the DADA2 plugin was applied for filtering, dereplication, chimera identification, and merging paired-end reads (Callahan et al., 2016) for further analysis. The alpha diversity indexes, including the Chao1 richness estimator, Shannon diversity, Simpson, and observed species, were calculated using the Amplicon Sequence Variant (ASV) table in QIIME2 and visualized as box plots. Beta diversity analysis was performed to investigate the structural variation of microbial communities across samples using Jaccard metrics (Jaccard, 1908) and visualized using principal coordinate analysis (PCoA). A Venn diagram was generated to visualize the shared and unique ASV among groups based on their occurrence, regardless of their relative abundance (Zaura et al., 2009). Taxa abundances at the ASV levels were statistically compared among samples or groups by MetagenomeSeq and visualized as Manhattan plots (Zgadzaj et al., 2016). Linear discriminant analysis effect size (LEfSe) was performed to detect differentially abundant taxa across groups using the default parameters (Segata et al., 2011). Co-occurrence network analysis was performed by SparCC analysis. The co-occurrence network was analyzed with the igraph package and visualized by Gephi 0.9.2 software.

### Statistical analysis

All data were displayed as mean ± standard deviation (SD). Statistical analysis was conducted with IBM SPSS Statistics 22 software (SPSS IBM Corp). Data variance was evaluated by one-way analysis. Means were compared by Duncan’s new multiple range test at the 5% level. Relationships between soil microbial ecological diversity and soil physical and chemical properties were analyzed by Pearson analysis. A *p*-value of <0.05 was considered statistically significant.

## Results

### Effects of straw-degrading microbes on soil’s physical and chemical properties

**Figure 1:**
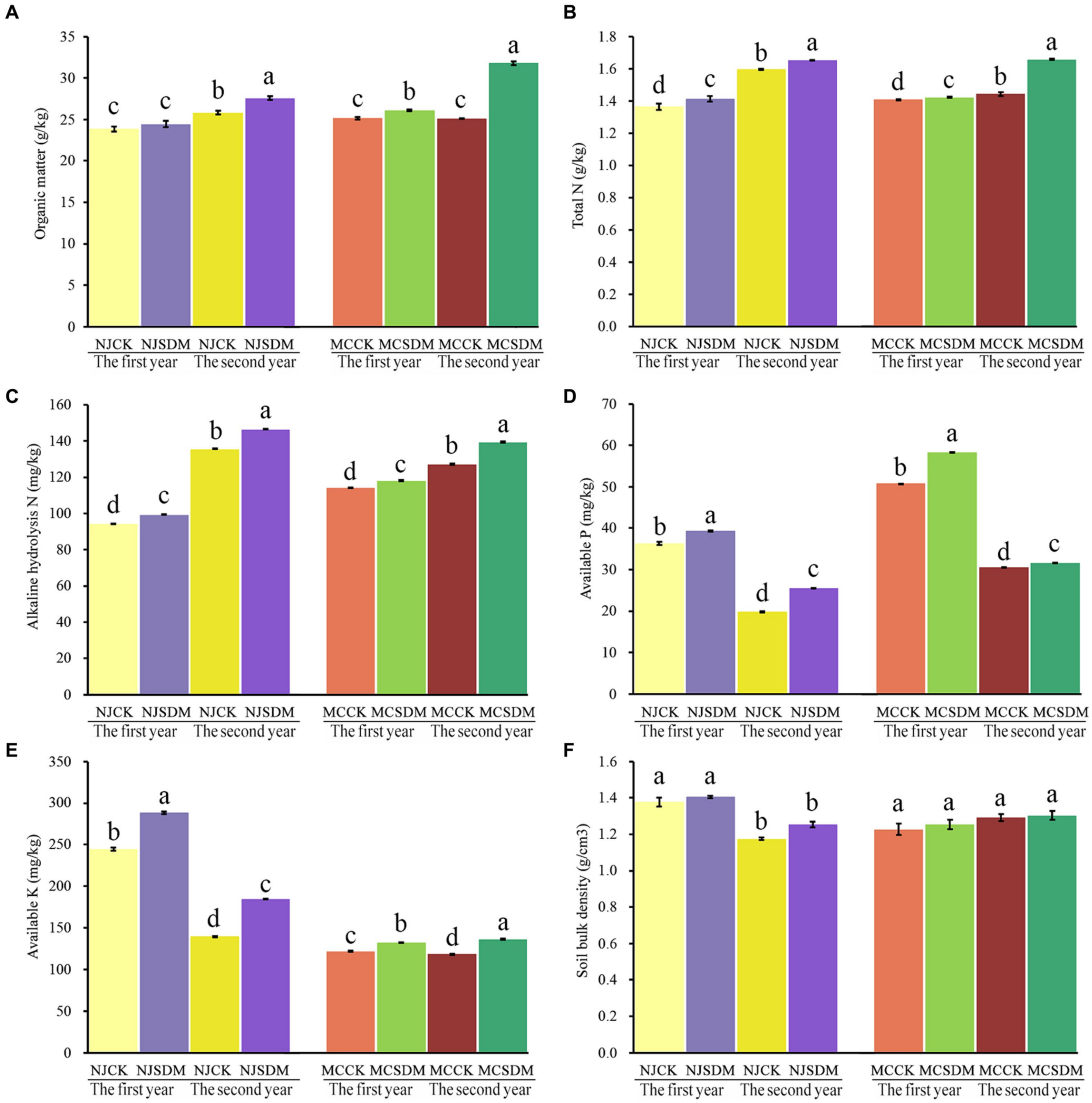
Effects of SDMs on soil physical and chemical properties, including organic matter **(A)**, total N **(B)**, alkaline hydrolysis N **(C)**, available P **(D)**, available K **(E)**, and soil bulk density **(F)**. The different lowercase letters stand for significant differences at a *p*-value of <0.05. CK is the control that the straw returned without SDMs in Ningjin (NJCK) and Mancheng (MCCK). The SDM is the treatment that the straw returned with the application of SDM in Ningjin (NJSDM) and Mancheng (MCSDM).

The application of SDMs in different soil textures significantly promoted soil chemical properties (Figures 1A–E). Two years of continuous application of the SDMs promoted the content of organic matter, total N, alkaline hydrolysis N, available P, and available K by 26.39, 15.32, 9.66, 3.94, and 15.19%, respectively, in SLS. In MLS, the content of organic matter, total N, alkaline hydrolysis N, available P, and available K was increased by 6.82, 3.62, 7.88, 28.39, and 32.26%, respectively. Soil C and N are the crop nutrition sources that were increased much higher in SLS than in MLS, and the P and K that promote crop development increased much higher in MLS compared with SLS. These results revealed that the SDMs significantly improved the soil nutrients, especially the SLS. The soil bulk density of MLS and SLS with the application of SDMs was higher than that of CK in the second year, but the difference was not significant (Figure 1F). These results suggested that the continuous application of SDMs with straw return would improve soil quality, especially for barren soil.

### Effects of straw-degrading microbes on wheat growth

**Figure 2:**
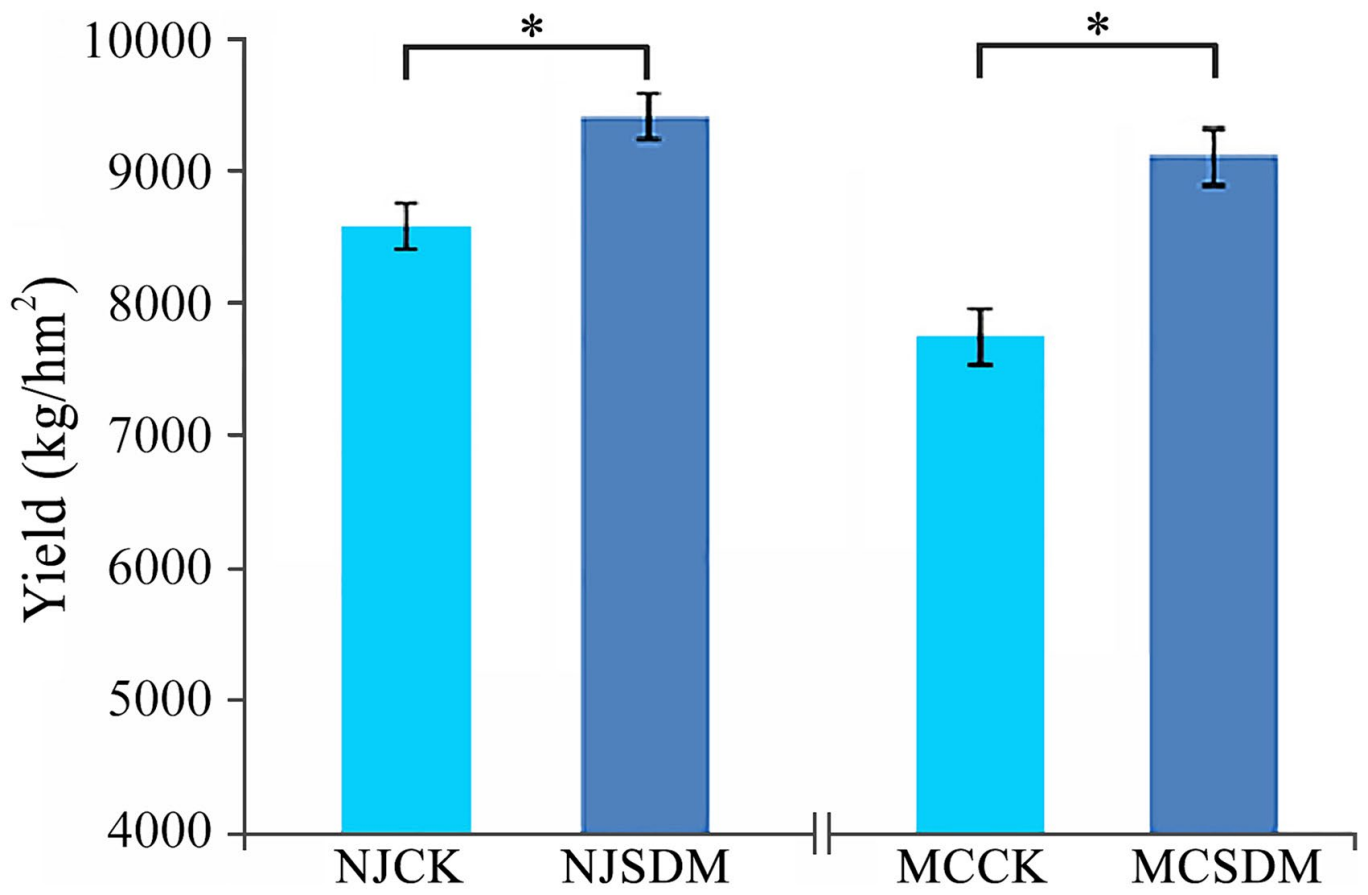
Effects of SDMs on the yield of winter wheat in the second year. Asterisks refer to significant differences at a *p*-value of < 0.05 level.

Changes in soil chemical and biological properties can significantly affect crop yield. Thus, the winter wheat yield was evaluated in 2023. The yield traits of wheat were higher in the SDM groups in both of the regions, including the numbers of spikes, grains per spike, and weight of 1,000 grains (Supplementary Figure S2). Although the yield traits that measured were not significantly varied, the total yield of winter wheat was significantly higher with the application of SDMs in both farmlands (Figure 2). The yield of winter wheat was increased by 17.4 and 9.5%, respectively, in MC and NJ after the application of SDMs. Thus, the compound SDM was effective in promoting the yield of winter wheat which were grown on soils with different textures.

### Effects of straw-degrading microbes on the α-diversity of soil microbial community

**Figure 3:**
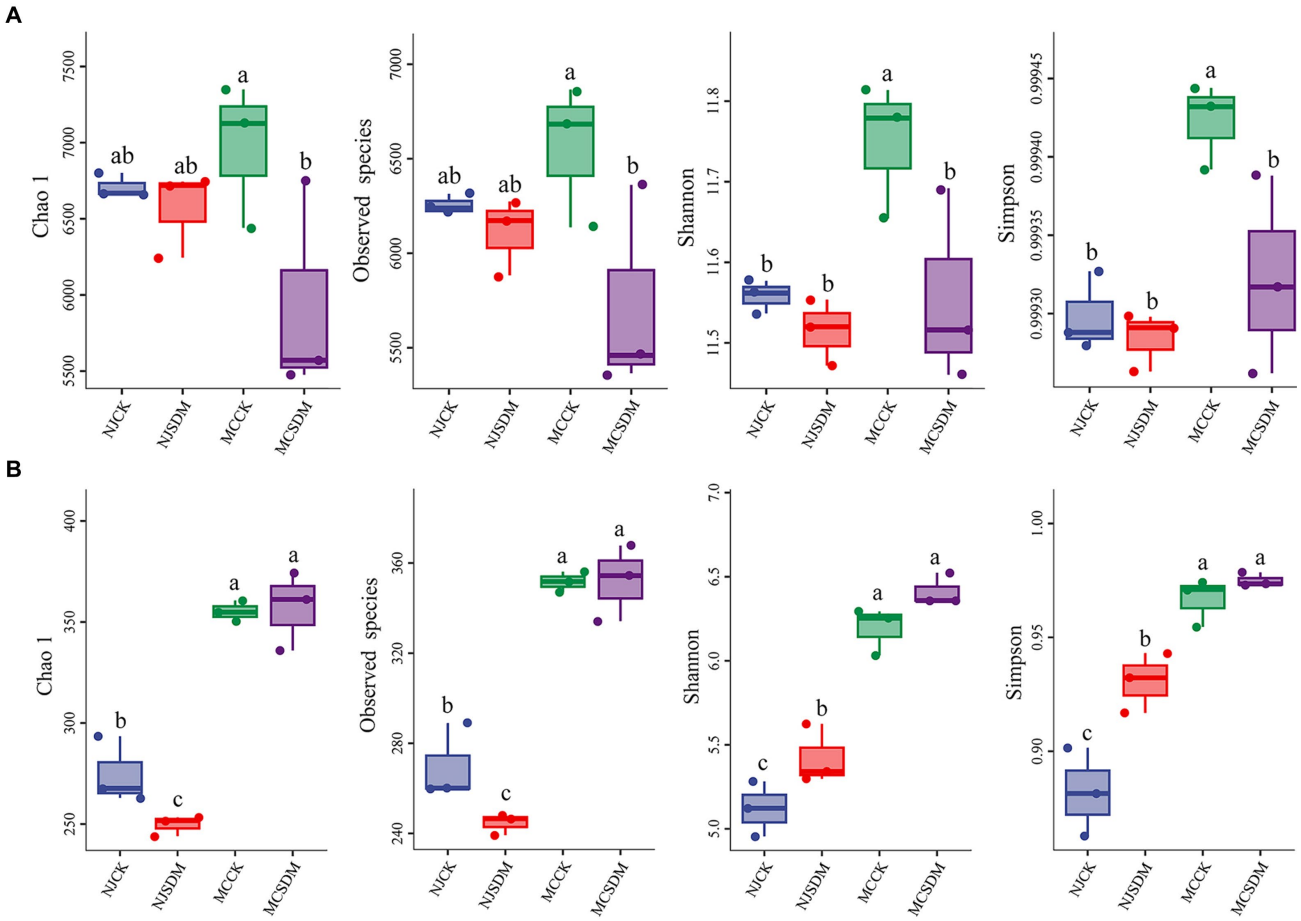
α-diversity of soil bacteria and fungi among the treatments in two experimental sites in the second year. **(A)** α-diversity of soil bacteria. **(B)** α-diversity of soil fungi. The different lowercase letters stand for significant differences at a *p*-value of <0.05 level.

Plants, animals, and microbes are the main components of the agricultural ecosystem. In order to investigate the effects of microbes on wheat after the application of SDMs, we analyzed the soil microbial diversity through sequencing. We assessed a total of 461,206 and 374,949 sequences (average 115,302 and 93,737 per sample) for bacteria and fungi, respectively. The results of *a-*diversity analysis demonstrated that the bacterial diversity and richness of the soils in different places were significantly different from each other (Figure 3A). The application of SDMs in the SLSs significantly decreased the bacterial Chao1, Shannon, and Simpson indexes and observed species. In contrast, the opposite results were obtained from the soil fungi (Figure 3B). The Chao1, Shannon, and Simpson indexes and observed species of fungi were only a little higher in the SLSs applied with SDMs. The Shannon and Simpson indexes of fungi were significantly more abundant with the application of SDMs in NJ, but the Chao1 and observed species indexes were lower. These results suggested that soil texture could affect soil microbial diversity, and the effects of the application of SDMs on soil microbial diversity might be determined by soil texture.

### Effect of straw-degrading microbes on the β-diversity of soil microbial community

The PCoA analysis showed that the three repeated microbial communities in different treatments all converged in one area and separated from the other treatments, suggesting the consistency of the replications. The samples from NJ were distributed far from the MC samples, suggesting great differences between the two soils (Supplementary Figure S3). The total variance explained by bacterial (PCo1) and fungal (PCo2) communities was 42.4 and 65.4%, respectively, between the four experimental groups, which suggested a greater effect of the SDMs on fungal diversity. The bacterial and fungal communities showed greater differences in MC, which was consistent with the α-diversity analysis.

### A Venn diagram analysis of OTUs by applying straw-degrading microbes

**Figure 4:**
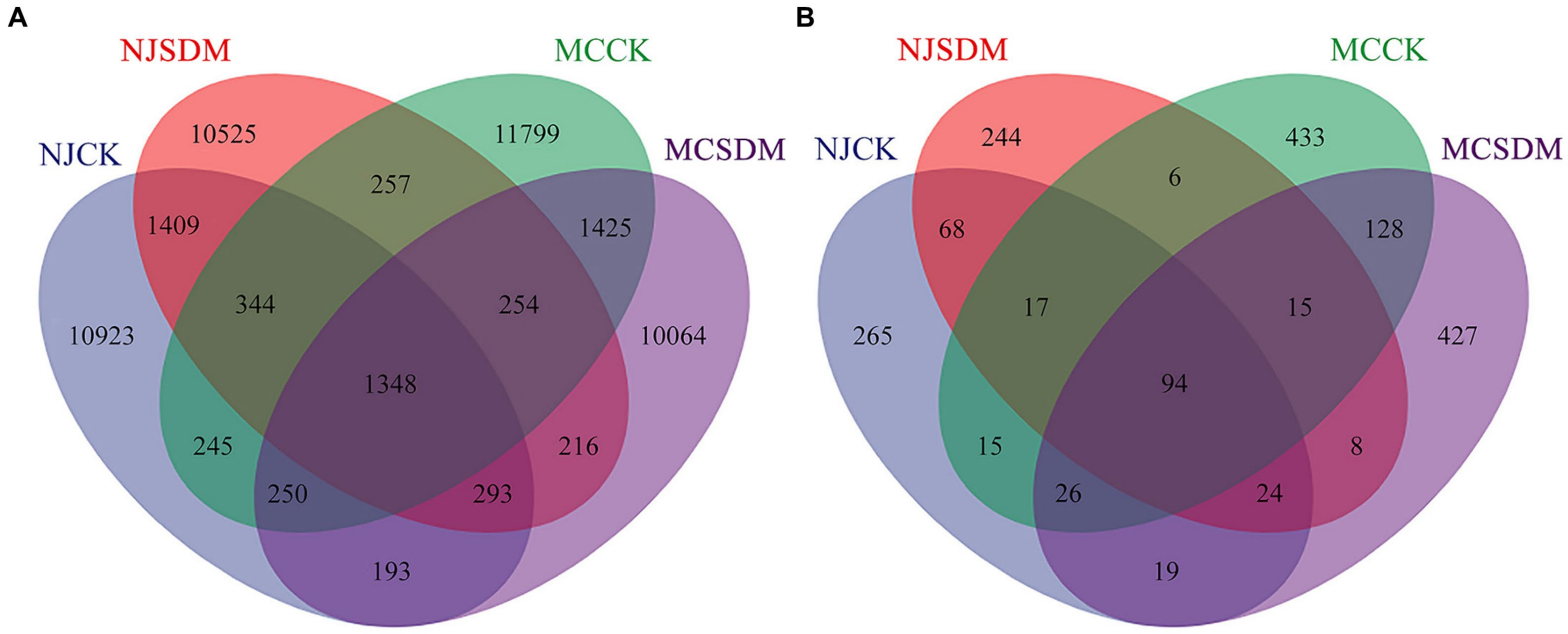
Venn diagrams of the OTU distribution of the 16S rRNA gene **(A)** and the ITS gene **(B)**.

A Venn diagram was used to compare the OTUs in the four groups. A total of 2.72% (1348) of the bacterial OTUs and 5.25% (94) of the fungal OTUs were shared in the four experimental groups (Figure 4). The specific bacteria in each group took 77.38% (11611), 76.38% (11252), 79.42% (12645), and 76.66% (10766) of the NJCK, NJSDM, MCCK, and MCSDM OTUs, respectively (Supplementary Figures S3A,B). While the specific fungi in each group took 61.55% (325), 78.15% (273), 64.16% (471), and 64.51% (478) of the NJCK, NJSDM, MCCK, and MCSDM OTUs, respectively (Supplementary Figures S4C,D). These results showed that the unique bacterial OTU composition was reduced, while the unique fungal OTUs were increased by applying SDMs.

### Microbial community structure and composition

**Figure 5:**
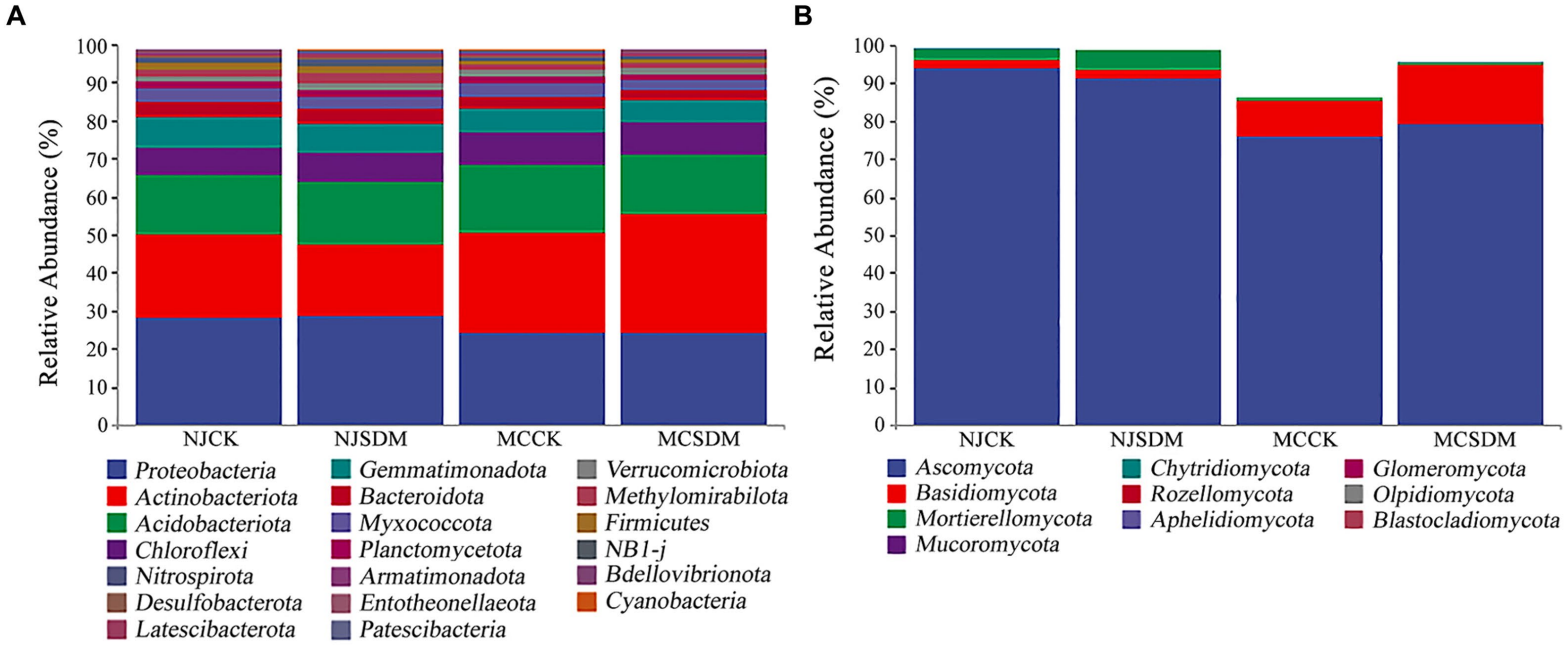
Microbial composition at the phylum level. **(A)** The 16S rRNA gene-based bacterial community composition. **(B)** The ITS gene-based fungal community composition.

Furthermore, the microbial community structure was investigated at the phylum level. The predominant bacterial phyla were Proteobacteria, Actinobacteriota, Acidobacteriota, Chloroflexi, and Gemmatimonadota, with a relative abundance above 5% (Figure 5A). Proteobacteria was the most dominant bacterial phylum in NJ. However, the most dominant bacterial phylum in MC was Actinobacteriota. The application of the SDMs increased the relative abundance of Actinobacteriota in SLS. The dominant fungal phylum was Ascomycota in all the experimental groups (Figure 5B). The SDMs increased the relative abundance of Mortierellomycota in NJ, while increased the relative abundance of Basidiomycota in MC.

**Figure 6:**
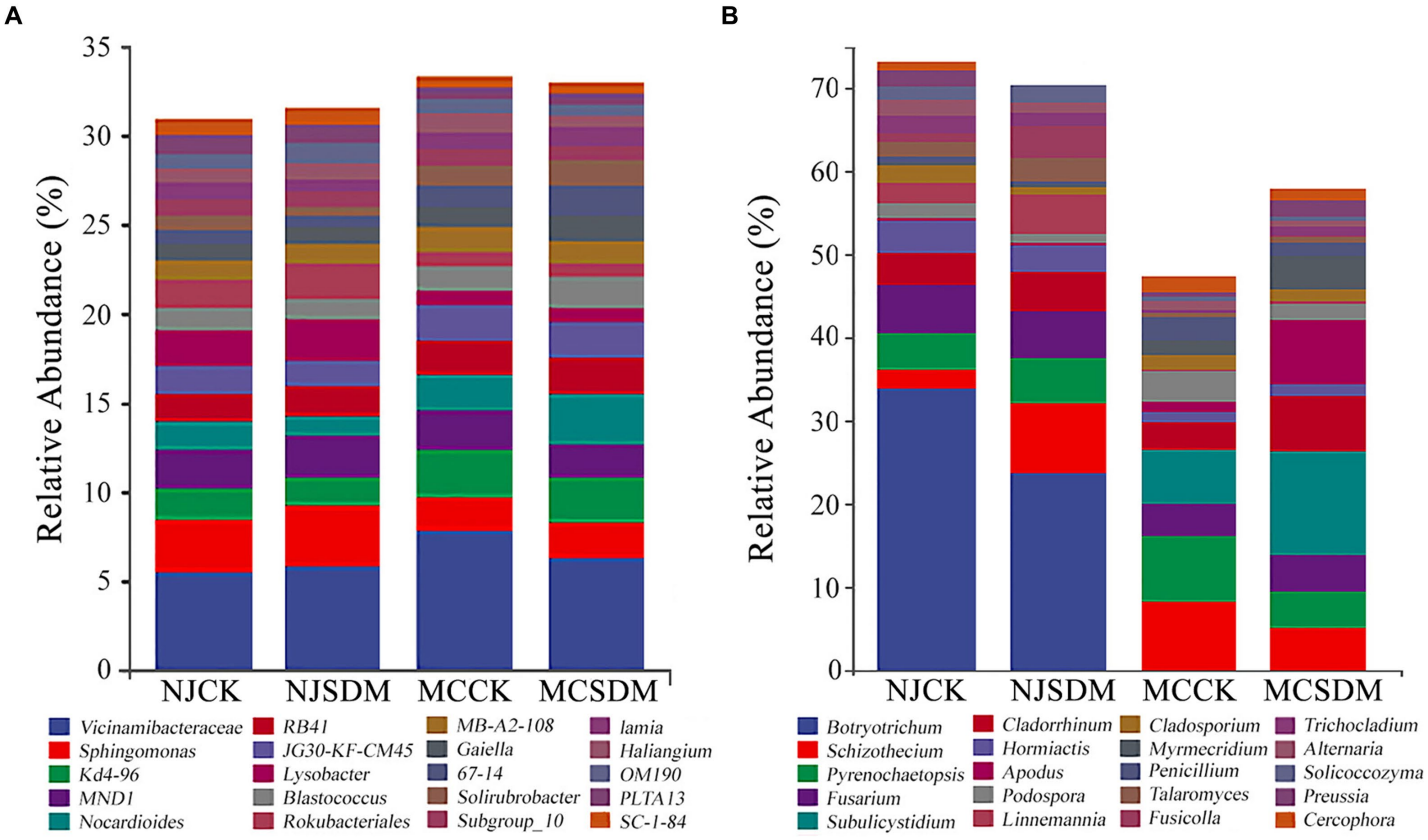
Microbial composition at the genus level. **(A)** The 16S rRNA gene-based bacterial community composition. **(B)** The ITS gene-based fungal community composition. The top 20 genera of bacteria and fungi are shown in different colors.

Subsequently, a genus-level comparison was made between the microbial communities of the experimental groups. The genera type with relative abundance above 1% was the same, while the ratio of each genus varied between the two experimental places. The bacterial genus Vicinamibacteraceae was commonly abundant in all the groups with the highest relative abundance above 5% (Figure 6A). The application of SDMs could increase the relative abundance of Vicinamibacteraceae in MLS, but decrease in SLS. Furthermore, the application of SDMs could also decrease the relative abundance of *Sphingomonas*. At the genus level of fungus, the preponderant fungal genus was quite different in the two experimental places (Figure 6B). In NJ, *Botryotrichum* was the most dominant and SDMs increased the relative abundance of *Schizothecium* to lower the ratio of the dominant fungal genus. In MC, the SDMs greatly increased the abundance of the genus *Subulicystidium* (from 6.55 to 12.38%), *Cladorrhinum* (from 3.20 to 6.77%), and *Apodus* (from 1.23 to 7.72%). The above results suggested different variations in the bacterial and fungal community structures in different soil textures after applying SDMs.

### Effect of straw-degrading microbes on the compositions of bacterial and fungal biomarkers

**Figure 7:**
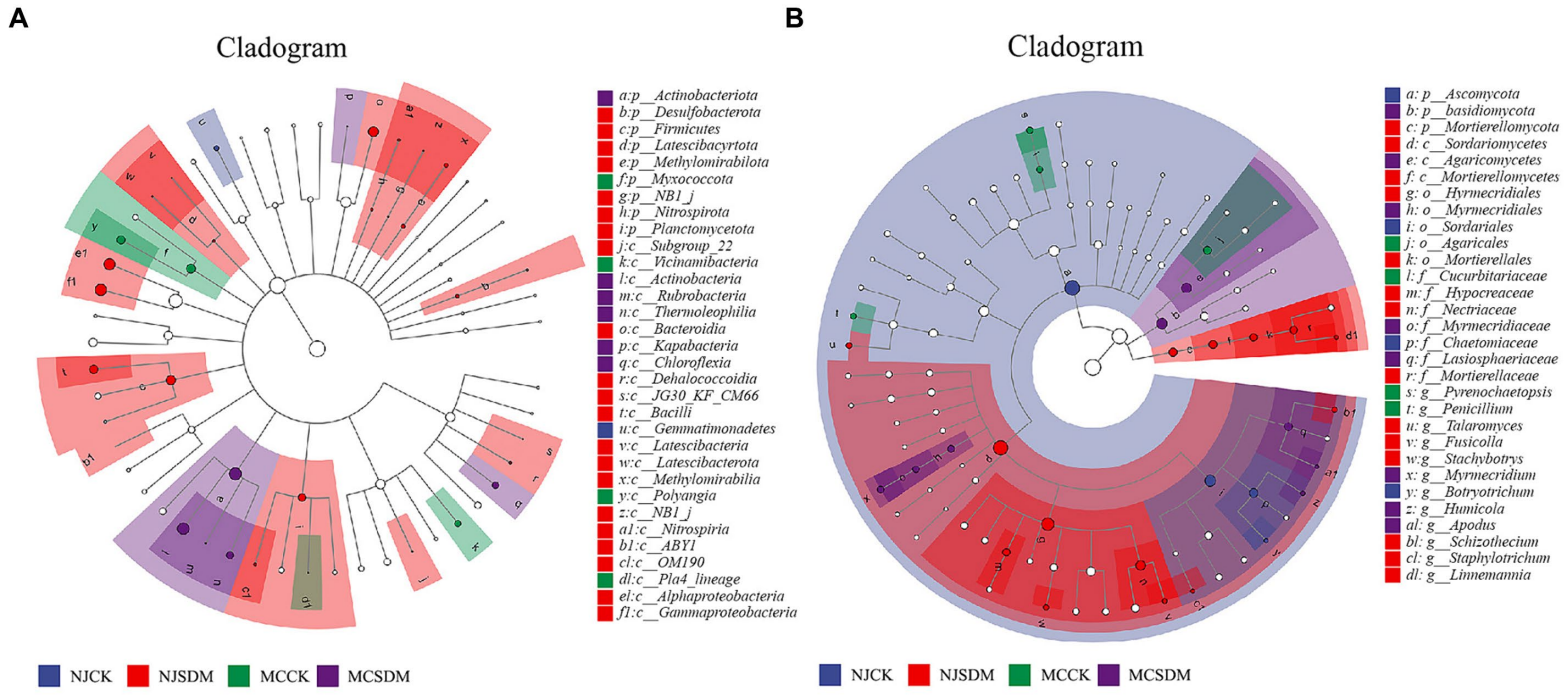
Cladograms plotted from the LEfSe analysis show significant differences (*p* < 0.05) in the relative abundance of 16S rRNA gene-based bacterial taxa **(A)** and ITS gene-based fungal taxa **(B)**.

LEfSe was applied to identify the bacterial and fungal community biomarkers (*p* < 0.05). Apart from the differences at phylum and genus levels, there were also other groups of microorganisms at the class, order, family, and genus levels (bacteria and fungi) that were enriched in the four experimental groups (Figure 7). Gemmatimonadetes (from class to order) were significantly enriched in NJCK. Two groups of Alphaproteobacteria and Gammaproteobacteria were significantly enriched in NJSDM. Three groups of bacteria, namely, Myxococcota, Vicinamibacteria (from class to order), and Polyangia, were significantly enriched in MCCK. Actinobacteriota (from phylum to class) were significantly enriched in MCSDM (Figure 7A). Four groups of fungi were significantly enriched in NJCK, namely, Chaetomiaceae, Botryotrichum, Sordariales, and Ascomycota. Sordariomycetes, Hypocreales, Nectriaceae, and Schizothecium were significantly enriched in NJSDM. Four fungal groups were enriched in MCCK, namely, Cucurbitariaceae, Pyrenochaetopsis, Penicillium, and Agaricales. Fungal groups, such as Agaricomycetes, Basidiomycota, and Lasiosphaeriaceae, were significantly enriched in MCSDM (Figure 7B).

### Effect of straw-degrading microbes on the co-occurrence network

**Figure 8:**
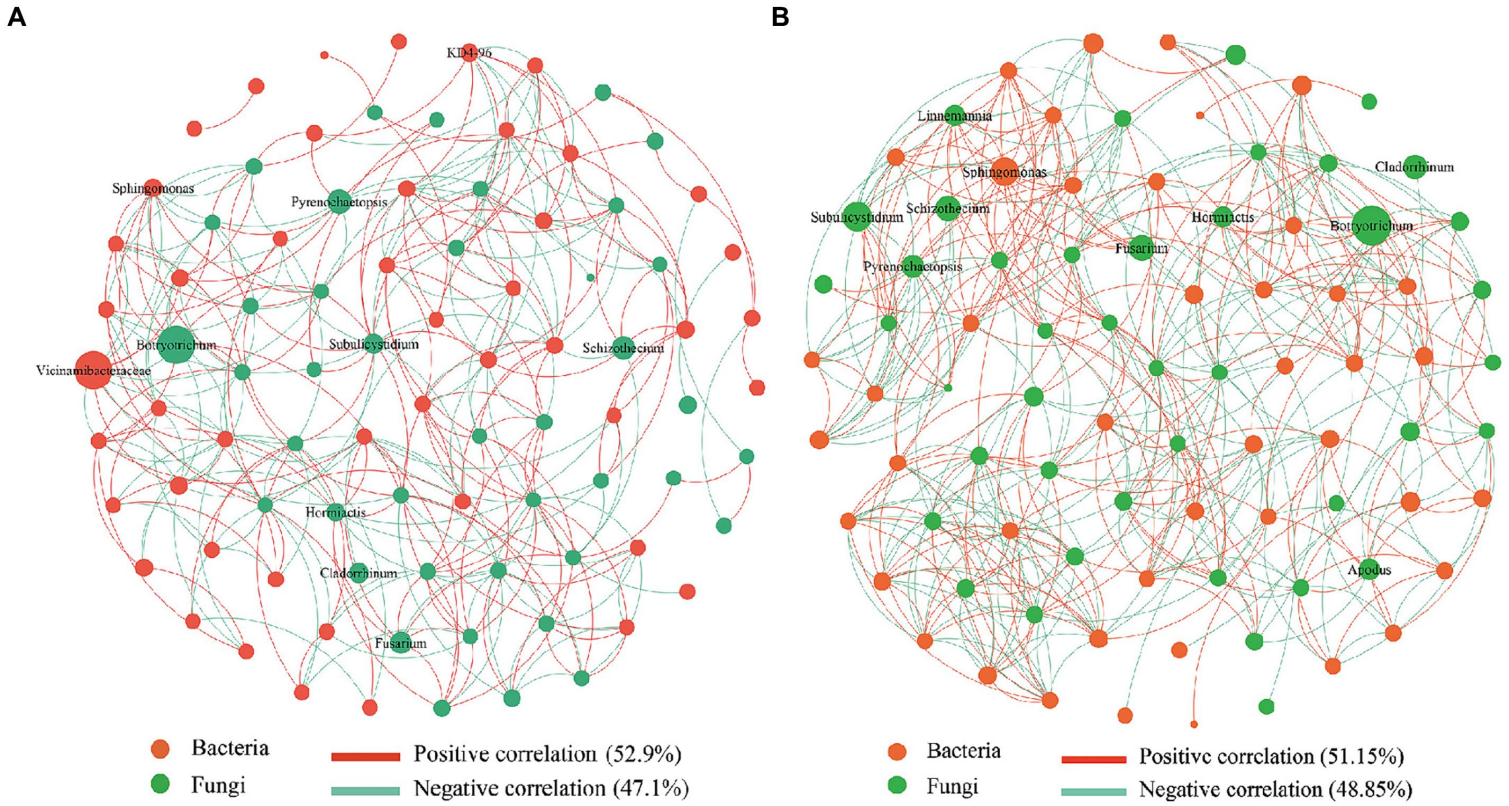
Co-occurrence network analysis of soil bacterial and fungal communities in the CK groups **(A)** and the SDM groups **(B)** of the two experimental sites. The size of each node is proportional to the relative abundance of the genus. Each connection shown has a Spearman’s coefficient of >0.6 and a *p*-value of <0.05. The red color represents bacteria, and the red line shows a positive correlation. The green color represents fungi, and the green line shows a negative correlation.

The symbiotic network relationship between the CK and SDM groups was obtained based on Spearman’s correlation coefficient. We found that bacteria dominated the symbiotic relationship among soil microbes and were the main components of the symbiotic systems (Figure 8). The dominant bacteria and fungi of CK groups were *Vicinamibacteraceae* and *Botryotrichum*, while the dominant bacteria of SDM groups was *Sphingomonas*. The correlation of bacteria decreased from 52.9 to 51.15%, while the correlation of fungi increased from 47.1 to 48.85%. The number of edges that indicate the complexity of microbial interaction increased by 40.65% in the SDMs compared with CK. The disease bacterium, *Fusarium,* displayed a more active correlation with the other microbes, indicating a limitation of its pathogenicity. These results suggested that the application of SDMs with straw changed the soil-dominant microbes and rebalanced their interactions, thus increasing their complexity, which is beneficial for crop growth.

### Correlation analysis between soil microbial diversity and soil physicochemical properties

Correlation analysis was performed between soil microbial diversity and environmental factors. From the soil bacterial correlation analysis (Table 2), we found that OM, TN, AN, and AK were negatively correlated with the Chao1, Shannon, and Simpon indexes and observed species. Significantly negative correlations were found between pH and AK with Shannon and Simpon indexes (*p* < 0.05). Furthermore, TN and AN were extremely significantly negatively correlated with Shannon and Simpon indexes (*p* < 0.01). In contrast, AP was positively correlated with Shannon and Simpon indexes. From the soil fungal correlation analysis (Table 3), OM was positively correlated with Chao 1, Shannon, and Simpon indexes and observed species. AP was extremely significantly positively correlated with Chao 1, Shannon, and Simpon indexes and observed species (*p* < 0.01). These results revealed that the changes in soil nutrients after the application of SDMs significantly affected the soil microbial richness and diversity.

**Table 2:** Correlation analysis between soil bacterial ecological diversity and soil chemical properties.

Factor	Chao1	Shannon	Simpon	Observed species
pH	0.070	−0.590*	−0.658*	−0.101
OM	−0.689	−0.373	−0.306	−0.645*
TN	−0.575	−0.781**	−0.785**	−0.660*
AN	−0.419	−0.756**	−0.768**	−0.534
AP	−0.191	0.413	0.517	−0.049
AK	−0.183	−0.656*	−0.674*	−0.322

OM, Organic matter; TN, total N; AN, alkaline hydrolysis N; AK, available K; AP, available P. ^*^p < 0.05; ^**^p < 0.01.

**Table 3:** Correlation analysis between soil fungal ecological diversity and soil chemical properties.

Factor	Chao1	Shannon	Simpon	Observed species
pH	−0.969**	−0.852**	−0.686*	−0.974**
OM	0.299	0.507	0.472	0.289
TN	−0.432	−0.228	−0.181	−0.445
AN	−0.621*	−0.319	−0.164	−0.631*
AP	0.781**	0.948**	0.960**	0.782**
AK	−0.795**	−0.500	−0.281	−0.803**

OM, Organic matter; TN, total N; AN, alkaline hydrolysis N; AK, available K; AP, available P. ^*^p < 0.05; ^**^p < 0.01.

## Discussion

Soil is a very diverse system consisting of various organic and inorganic components. One of the most essential constituents in the soil is microorganisms, which can degrade several compounds, resulting in a highly dynamic soil environment. Straw decomposition is a process of nutrient release, organic C mineralization, and soil organic C balance mediated by SDMs (Zhao et al., 2019; Mei et al., 2020), which promotes soil fertility, changes the number of soil microorganisms, and affects plant growth (Han and He, 2010; Said-Pullicino et al., 2014). Our research confirmed that applying SDMs significantly improved wheat yield, especially the wheat yield increased by 17.4% in the SLS of MC (Figure 2).

The SDMs promoted straw decomposition (Supplementary Figure S1), which improved soil chemical properties (Figure 1). The straw’s nutrients are immobilized, but the decomposition of the straw releases C and N into the soil. Organic matter and N determine soil fertility. The significant increase in the amount of SOM, total N, and available N revealed that the application of SDMs increased soil nutrient condition, which could significantly affect both crop yield and soil respiration (Chen et al., 2017; Liang et al., 2019, 2021). Phosphorus (P) is vital for plant growth and production, as it is required as an important part of the compounds of ATP (the energy carrier). P is necessary for starch and fat metabolism, cellular respiration, N uptake, and carbohydrate transportation (Adnan et al., 2020). Thus, the optimum level of P is important to obtain maximum yield (Cui et al., 2022). Studies proved that straw returning can increase the soil phosphorus supply capacity by reducing soil phosphorus adsorption and increasing soil phosphorus desorption capacity (Ding et al., 2024). Potassium (K) is another mineral element that is essential for plant growth and development and increases wheat yield (Zhan et al., 2016). Long-term straw returning is beneficial for soil K balance and K supply ability (Zhang et al., 2021). The straws are good sources of organic matter, plants N, P, and K. The straw degradation rate increased fast with the SDMs during the wheat jointing stage and the filling stage (Supplementary Figure S1), which not only provided nutrients for wheat but also improved yield traits at the main developmental stages, resulting in a significant increase in wheat yield.

Straw decomposition affects soil microorganisms whose diversity and community structure influence key soil chemical characteristics (Zhang et al., 2023). Different levels of straw degradation with different nutrient release capacities and metabolites create different soil biological environments (Kandasamy et al., 2019). Our results were consistent with this that SDM groups increased the fungi composition and diversity (Figures 3, 4). The coexistence of bacteria and fungi in soil forms a complex system of species interactions. During the decomposition process of straw, the dominant microbial communities in the early and late stages of decomposition are different (Bastian et al., 2009). Previous research showed that bacteria are the dominant microbes in the early stage of straw decomposition, while fungi will become dominant in the soil after active straw degradation (Bastian et al., 2009). In this study, the appropriate amount of SDMs was applied considering environmental safety so that the exogenous microbes were unable to ‘overturn’ the microbial community (Ho and Ko, 1985), which is consistent with the fact that *Pseudogymnoascus* was not dominant in the soil (Figure 6). The degradation of the straw by *Pseudogymnoascus* gradually changed the soil microbial community, in which fungal richness increased (Figure 3; Supplementary Figure S4). The bacterial diversity in SLS in MC was significantly affected by SDMs, but there was little difference in MLS in NJ. The fungal diversity only significantly varied in MLS in NJ. The same effects of different composition agents were found on SLS and MLS under straw return (Sa et al., 2020). Thus, it was speculated that the effects of SDMs on soil microbial diversity were related to the composition of straw decomposing agents and soil type.

The dominant bacterial phyla in the soil were Proteobacteria, Actinobacteriota, and Acidobacteria (Figure 5A), which are commonly found in soils worldwide (Maestre et al., 2015). The dominant fungal groups were Ascomycota and Basidiomycota (Figure 5B), which prevail in terrestrial ecosystems (Tedersoo et al., 2014). The relative abundance of the bacterial genus *Sphingomonas* in the Proteobacteria phylum increased with the application of SDMs (Figure 6). *Sphingomonas* can metabolize a wide variety of carbon sources and clean up toxic substances in soil. Some possess the characteristics of nitrogen fixation and denitrification (Yang et al., 2016). The members of Actinobacteriota produce extracellular hydrolytic enzymes to decompose organic matter and enhance soil carbon cycling. The relative abundance of dominant fungal phyla in MCSDM increased significantly. The ability of cellulose and lignin degradation is particularly common in Ascomycota and Basidiomycota (Hannula et al., 2010; Zhang et al., 2019; Wu et al., 2021; Zhao et al., 2021). Basidiomycota is positively correlated with soil C and N (Zhu et al., 2022). The changes in bacterial and fungal community structure supported the higher increase of soil C and N resulting in a more effective improvement of wheat yield in MC.

Combining the LEfSe analysis with microbial community composition, we found that Alphaproteobacteria and Gammaproteobacteria were significantly enriched in NJSDM, and Actinobacteriota was significantly enriched in MCSDM (Figure 7A), which may be caused by the soil particle size and nutrient contents. The growth of microorganisms is regulated by soil fraction size and nutrients (Sessitsch et al., 2001; Lu et al., 2020; Xia et al., 2020). The effects of soil type on soil microbial community structure are determined by different soil environmental factors. Our results displayed different variations of microbial community structure related to soil nutrients (Tables 2, 3). The soil pH is considered the main factor driving the changes in soil microbial diversity (Pennanen, 2001; Oehl et al., 2010). The vital activities of the microbial community are supported by soil nutrients. Total N and available N are negatively correlated with microbial diversity, while available P is positively correlated with soil microbial diversity (Tables 2, 3), which was supported by previous research (Yue et al., 2023). Additionally, the plant root exudates are decomposed and absorbed by soil microorganisms, which accelerate soil nutrient accumulation. The microbial community is essential for soil quality.

The microorganism is a part of the soil ecosystem, whose interaction is crucial for determining the function and stability of the entire ecological network. The key species with specific functions in the soil were changed by SDM (Figure 8B). The disease bacterium, *Fusarium,* displayed a more active correlation with the other microorganisms, indicating a limitation of its pathogenicity. The interactions with *Sphingomonas* were more complex. The clustering coefficient, graph density, and average degree of SDMs were all higher than those of CK, suggesting a more complex microbial network. Furthermore, the application of SDMs with straw return increased specific fungal OTUs and decreased specific bacterial OTUs (Figure 4; Supplementary Figure S4). This shift revealed a higher ratio of fungi/bacteria, suggested as an indicator for a more sustainable agroecosystem where organic matter decomposition and N mineralization dominate the nutrient supply for crop growth (De Vries et al., 2006), which is beneficial for crop reproduction. Additionally, a large proportion of Basidiomycota can form mycorrhizas with plant roots in the soil (Kumar and Atri, 2018). The increase in this phylum indicated interactions with wheat roots to promote wheat growth and development.

## Conclusion

The application of SDMs could significantly promote the soil’s chemical properties, increase wheat yield, and promote the microorganism environment in the soil. The density and complexity of the symbiotic network of SDMs were increased, and the community structure was more stable. Furthermore, the SDMs were found to be more effective in SLS, revealing a potential utilization for straw return in winter.

## Data availability statement

The datasets presented in this study can be found in online repositories. The names of the repository/repositories and accession number(s) can be found at: https://www.ncbi.nlm.nih.gov/, OM 304630.

## Author contributions

YuH: Conceptualization, Funding acquisition, Investigation, Methodology, Writing – original draft, Writing – review & editing. YY: Data curation, Formal analysis, Investigation, Writing – original draft, Writing – review & editing. YM: Formal analysis, Writing – review & editing. XZ: Formal analysis, Writing – review & editing. QZ: Formal analysis, Writing – review & editing. MM: Conceptualization, Supervision, Writing – review & editing. YaH: Conceptualization, Supervision, Writing – review & editing. ZP: Conceptualization, Supervision, Writing – review & editing.

## Supplementary material

SUPPLEMENTARY FIGURE S1 The degradation rate of corn straw at different developmental stages of wheat. CK is the control that the straw returned without straw-degrading microbes. SDM stands for experimental group that the straw returned with application of the straw-degrading microbes.

SUPPLEMENTARY FIGURE S2 Effects of SDM on traits of wheat yield including spike number **(A)**, number of grains per spike **(B)**, and 1000-grain weight **(C)**.

SUPPLEMENTARY FIGURE S3 Effect of SDM on β-diversity of soil microbial community. Principal coordinate analysis (PCoA) of the bacterial 16S rRNA gene **(A)** and fungal ITS gene **(B)**. Each colored dot represents the sample.

SUPPLEMENTARY FIGURE S4 Venn diagrams of OTU distribution of the 16S rRNA gene **(A,B)** and the ITS gene **(C,D)** among CK and SDM treatments in two experimental sites.
