# Supplementary figures and images for: The effect of low-temperature straw-degrading microbes on winter wheat growth and soil improvement under straw return
Source: Front Microbiol. 2024 Jul 11;15:1391632. doi: 10.3389/fmicb.2024.1391632 (PMC11269160; doi:10.3389/fmicb.2024.1391632)

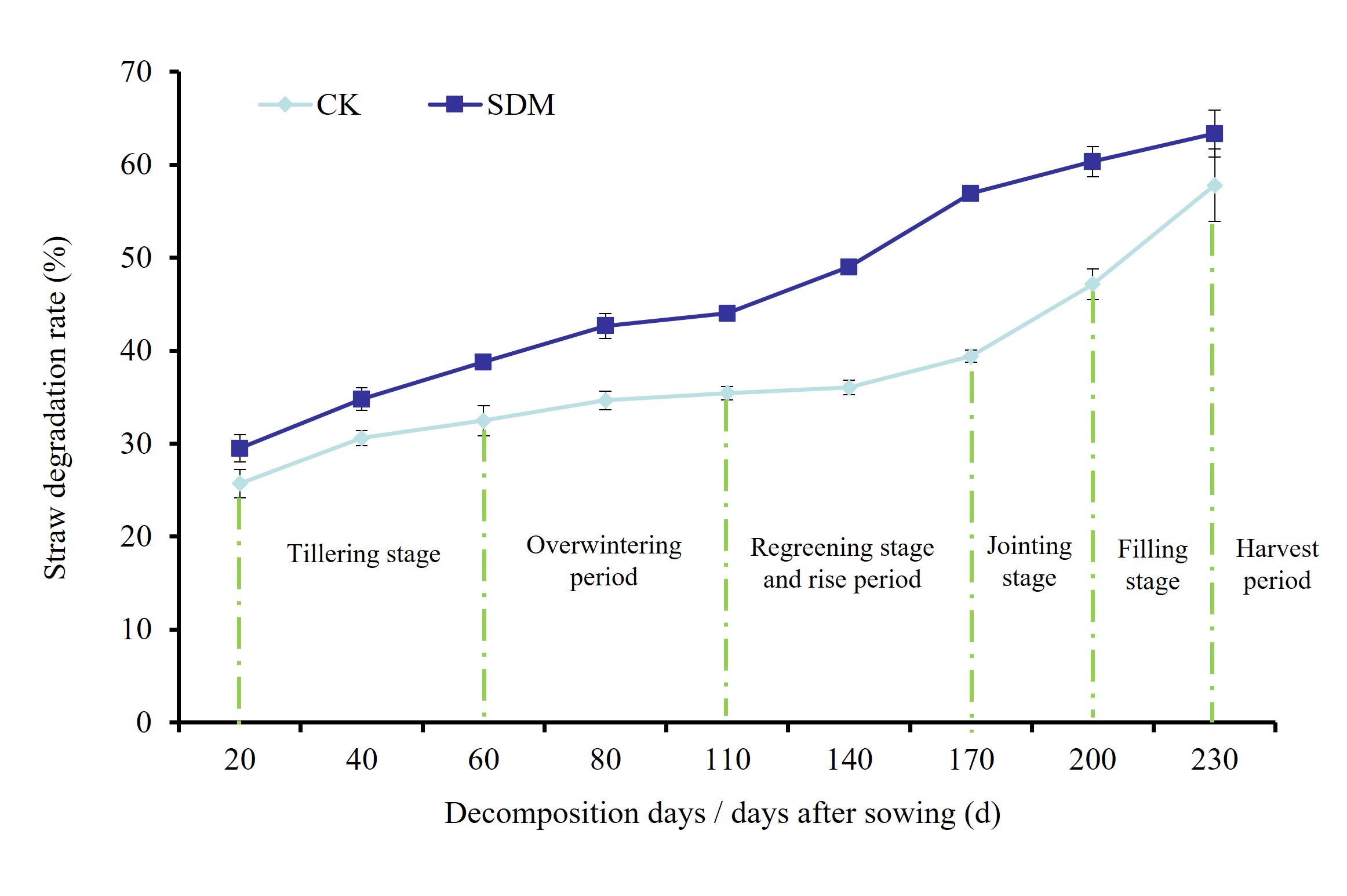

Supplement: SUPPLEMENTARY FIGURE S1 — The degradation rate of corn straw at different developmental stages of wheat. CK is the control that the straw returned without straw-degrading microbes. SDM stands for experimental group that the straw returned with application of the straw-degrading microbes. [file Image_1.JPEG]

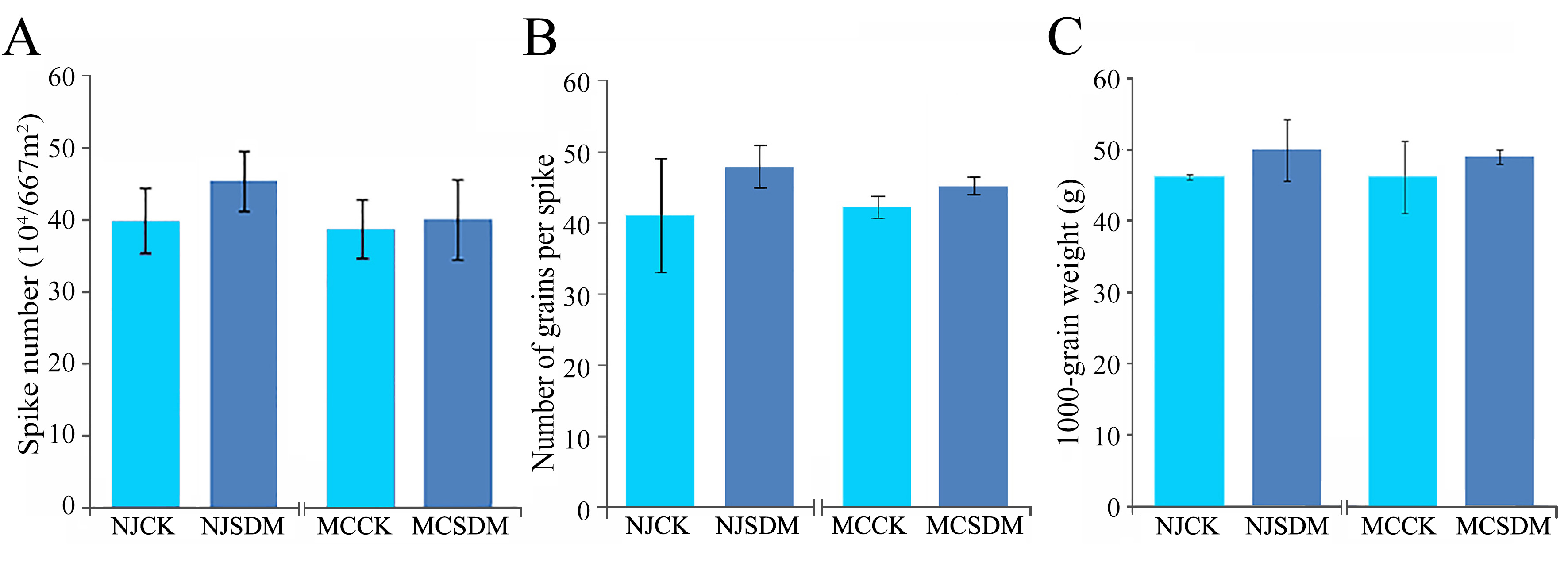

Supplement: SUPPLEMENTARY FIGURE S2 — Effects of SDM on traits of wheat yield including spike number (A), number of grains per spike (B), and 1000-grain weight (C). [file Image_2.JPEG]

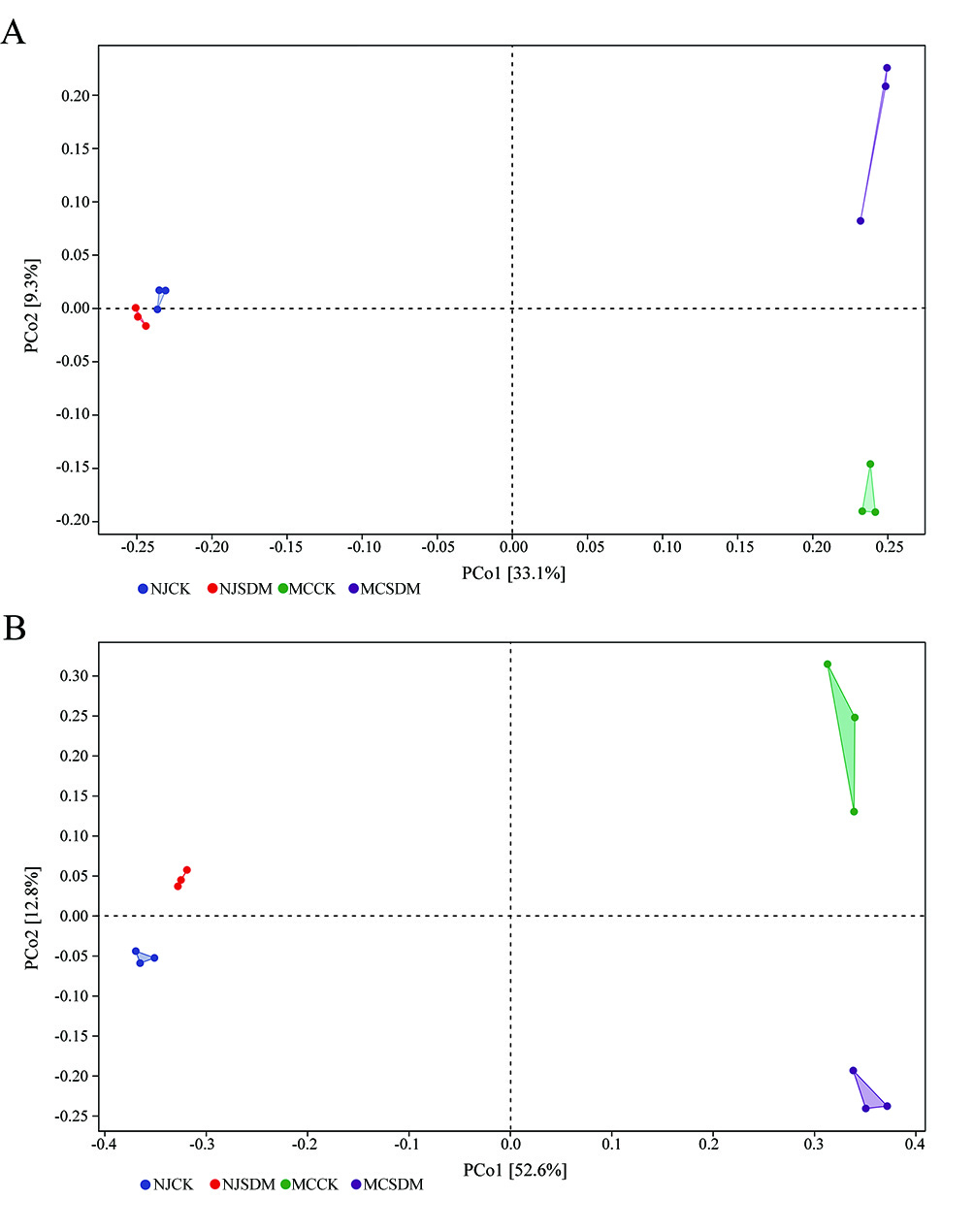

Supplement: SUPPLEMENTARY FIGURE S3 — Effect of SDM on β-diversity of soil microbial community. Principal coordinate analysis (PCoA) of the bacterial 16S rRNA gene (A) and fungal ITS gene (B). Each colored dot represents the sample. [file Image_3.JPEG]

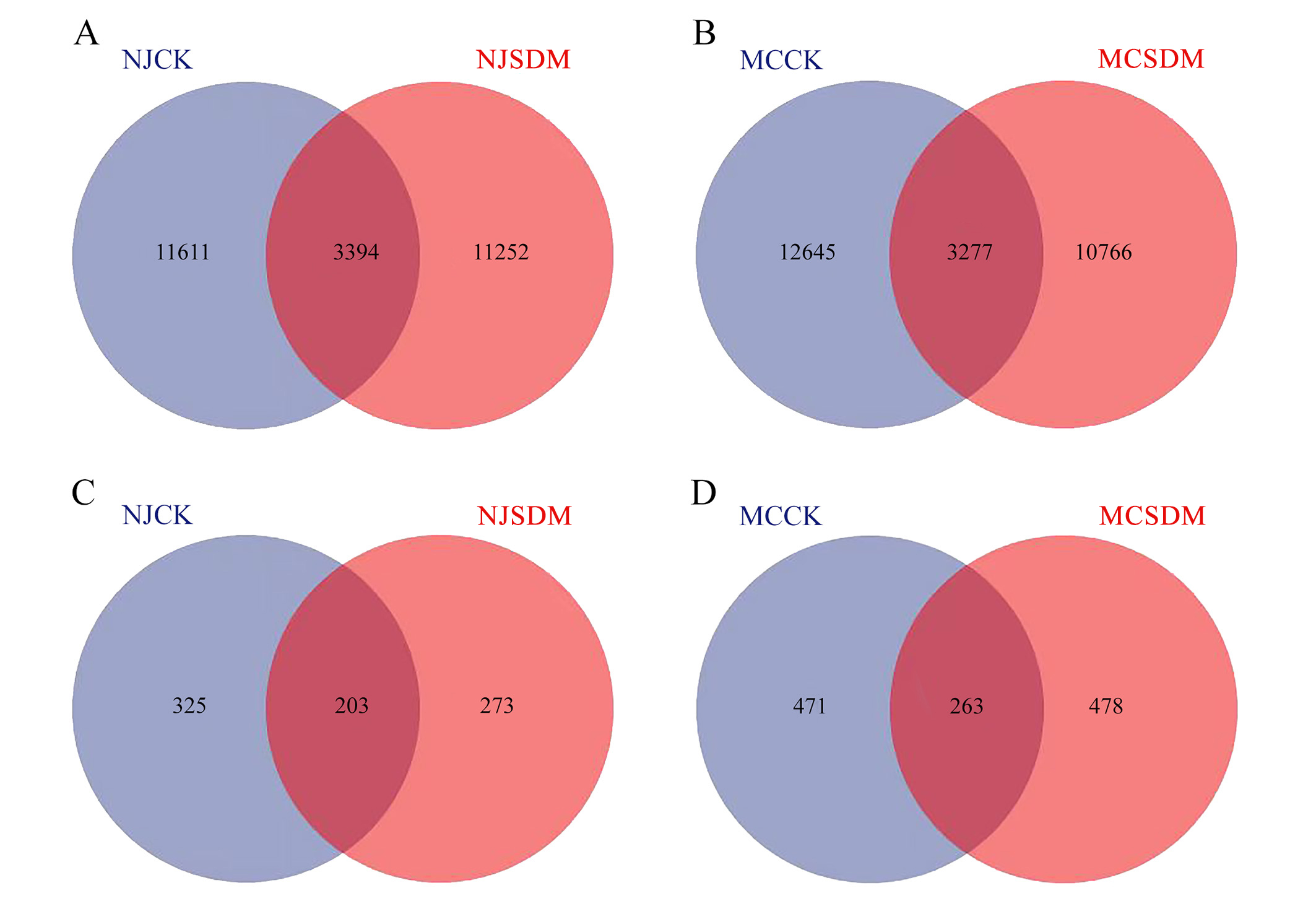

Supplement: SUPPLEMENTARY FIGURE S4 — Venn diagrams of OTU distribution of the 16S rRNA gene (A,B) and the ITS gene (C,D) among CK and SDM treatments in two experimental sites. [file Image_4.JPEG]
